# Multi-PK antibodies: Powerful analytical tools to explore the protein kinase world

**DOI:** 10.1016/j.bbrep.2017.06.005

**Published:** 2017-06-22

**Authors:** Yasunori Sugiyama, Isamu Kameshita

**Affiliations:** Department of Life Sciences, Faculty of Agriculture, Kagawa University, Kagawa 761-0795, Japan

**Keywords:** 2D-PAGE, two-dimensional polyacrylamide gel electrophoresis, CaMK, Ca^2+^/calmodulin-dependent protein kinase, CDKL5, cyclin-dependent kinase-like 5, CNBr, cyanogen bromide, DCLK, double-cortin like protein kinase, Dnmt1, DNA methyltransferase 1, FAK, focal adhesion kinase, IEF, isoelectric focusing, IPG, immobilized pH gradient, MAPK, mitogen-activated protein kinase, MeCP2, methylated-CpG-binding protein 2, Protein kinase, Monoclonal antibody, Protein phosphorylation, Proteomics, Kinomics

## Abstract

Diverse biological events are regulated through protein phosphorylation mediated by protein kinases. Some of these protein kinases are known to be involved in the pathogenesis of various diseases. Although 518 protein kinase genes were identified in the human genome, it remains unclear how many and what kind of protein kinases are expressed and activated in cells and tissues under varying situations. To investigate cellular signaling by protein kinases, we developed monoclonal antibodies, designated as Multi-PK antibodies, that can recognize multiple protein kinases in various biological species. These Multi-PK antibodies can be used to profile the kinases expressed in cells and tissues, identify the kinases of special interest, and analyze protein kinase expression and phosphorylation state. Here we introduce some applications of Multi-PK antibodies to identify and characterize the protein kinases involved in epigenetics, glucotoxicity in type 2 diabetes, and pathogenesis of ulcerative colitis. In this review, we focus on the recently developed technologies for kinomics studies using the powerful analytical tools of Multi-PK antibodies.

## Highlights

• Multi-PK antibodies recognize a wide variety of protein kinases.
• New analytical methods using Multi-PK antibodies for protein kinase studies are explained.
• Kinomics studies using Multi-PK antibodies are introduced.

## 1 Introduction

**Fig. 1:**
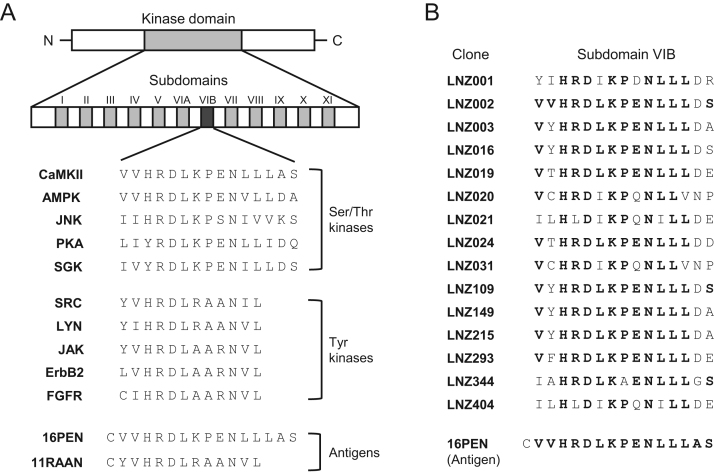
Amino acid sequences of the subdomain VIB and the peptides used as antigens for the generation of Multi-PK antibodies. (A) Alignment of subdomain VIB sequences in various protein kinases and the peptides used as antigens: 16PEN for M1C and M8C antibody production; 11RAAN for YK34 antibody production. (B) Amino acid sequences of subdomain VIB in Ser/Thr protein kinases in plant. The clones for Ser/Thr kinases were isolated from a cDNA library of *L*. *japonicus* by expression screening using the M1C/M8C antibodies.

Protein kinases play important roles in various biological phenomena through the regulation of phosphorylation signaling pathways [1]. Eukaryotic protein kinases make up a large superfamily of homologous proteins, comprising 1.5–2.5% of all gene products [2]. Genome projects have been completed for various species, and as many as 518 protein kinase genes were identified in the human genome [3]. These enzymes are classified into two major groups: Ser/Thr protein kinases and Tyr protein kinases. Although these enzymes have different sizes, isoelectric points, substrate specificities, and regulatory mechanisms, they share a homologous catalytic core. As shown Fig. 1A, the kinase domain, consisting of 250–300 conserved amino acid residues, can be divided into 12 subdomains that contain essential sequences for the structural features required for protein kinase catalytic activities [4].

A variety of protein kinases are known to be involved in biological phenomena such as proliferation, development, differentiation, and apoptosis through protein phosphorylation [2]. To investigate the protein kinases involved in these events, various tools and methods for analysis of cellular kinases have been developed. For expression analyses, Western blotting with protein kinase-specific antibodies is widely used for evaluation of the protein expression levels of protein kinases [5], [6], [7], [8], while real-time PCR is used for examination of the mRNA expression levels [9]. Recently, a cDNA microarray was employed for analysis of the entire RNA expression levels of protein kinases [10]. Meanwhile, for activity analyses, protein and peptide substrates are used for *in vitro* kinase assays to determine the protein kinases activities [11], [12], [13], [14], and Western blotting analysis with phosphorylation site-specific antibodies is widely used to assess the activation states [5], [6], [7], [8]. However, a method that can simultaneously analyze the protein expression and/or activity levels of the entire protein kinases in cells has not been established, because of the lack of specific probes to globally detect multiple protein kinases. It is important to analyze the expression profiles of the entire protein kinases under varying situations to elucidate the cellular signaling mechanisms. Based on these backgrounds, we have produced monoclonal antibodies, designated as Multi-PK antibodies, to analyze a wide variety of protein kinases [15], [16]. In this review, we introduce the process for the generation of Multi-PK antibodies, the methods for analysis of protein kinases using Multi-PK antibodies, and the applications of Multi-PK antibodies to explore phosphorylation signaling.

## 2 Multi-PK antibodies

In general, it is widely recognized that a superior antibody has strict specificity for its specific antigen and does not exhibit nonspecific cross-reactivity. However, we hypothesized that an antibody with broad specificity could be useful for the analysis of diverse protein kinases simultaneously. The main structural feature of the protein kinases families of enzymes is a catalytic core consisting of 12 highly conserved subdomains [4]. While subdomain VIB appears to be the most highly conserved region among the 12 subdomains in many protein kinases, the sequences of this region in Ser/Thr kinases differ somewhat from those in Tyr kinases. The typical subdomain VIB sequences of Ser/Thr kinases are H-R-D-L-K-P-(E/S)-N, while those of Tyr kinase are H-R-D-L-(R/A)-A-(A/R)-N (Fig. 1A) [15], [16]. Based on these findings, we synthesized antigenic peptides,16PEN (CVVHRDLKPENLLLAS) and 11RAAN (CYVHRDLRAANVL), corresponding to the subdomain VIB sequences of Ser/Thr kinases and Tyr kinases, respectively, and used these peptides for immunization of BALB/c mice. As a result, we established three hybridoma cell lines (M1C, M8C, YK34) producing monoclonal antibodies, Multi-PK antibodies, with broad cross-reactivities [15], [16]. Specifically, the M1C and M8C antibodies recognize Ser/Thr kinases and the YK34 antibody detects Tyr kinases.

### 2.1 Cross-reactivities of Multi-PK antibodies

To investigate the immunoreactivities of the M1C and M8C antibodies, cDNA expression libraries of the mouse brain [15], *Xenopus laevis* embryo [17], *Lotus japonicus* root nodule [18], basidiomycete mushroom *Coprinopsis cinerea* [19], and zebrafish *Danio rerio* [20] were immunologically screened with the M1C and M8C antibodies. Among the many positive clones obtained using these antibodies, nearly 93% turned out to be Ser/Thr protein kinases. The amino acid sequences of subdomain VIB in the Ser/Thr kinases isolated from *L*. *japonicus* are shown in Fig. 1B. When Western blot analyses were carried out using the M1C and M8C antibodies, Ser/Thr kinases, such as Ca^2+^/calmodulin-dependent protein kinase (CaMK) I [21], CaMKII, CaMKIV, CaMK kinase, cAMP-dependent protein kinase [15], cyclin-dependent kinase-like 5 (CDKL5) [22], doublecortin-like protein kinase (DCLK) [23], nuclear dbf2-related kinase [24], Akt, c-Jun N-terminal protein kinase 1, mitogen-activated protein kinase (MAPK), MAPK kinase, and microtubule affinity-regulating kinase, were detected. The results revealed that the M1C and M8C antibodies recognized Ser/Thr protein kinases with subdomain VIB sequences of (H/Y)-(R/L)-D-(L/V/I)-K-(P/A)-(E/D/Q/S)-N.

To examine the cross-reactivity of the YK34 antibody, we employed different SRC Tyr kinase recombinants with various amino acid replacements in subdomain VIB. By Western blotting analysis, we found that the YK34 antibody recognized the amino acid sequences (Y/F/L/C)-(V/I)-H-R-D-L-(R/A)-(A/T)-(A/R)-N [16]. Indeed, Tyr kinases such as SRC, SYK, ABL, LYN [16], [25], and focal adhesion kinase (FAK) [26] were detected by the YK34 antibody. Taking these results into consideration, we can speculate that YK34 antibody would recognize more than 75% of Tyr kinases.

Taken together, these findings suggest that Multi-PK antibodies can be powerful tools to detect Ser/Thr kinases and Tyr kinases in proteomics studies focused on a wide range of protein kinases (kinomics).

## 3 New analytical methods using Multi-PK antibodies

Multi-PK antibodies can be used for Western blotting and immunoprecipitation of various protein kinases [15], [16]. Multiple protein kinases can be detected in crude cell extracts from various biological species using Multi-PK antibodies. In this section, we introduce newly developed analytical methods using Multi-PK antibodies: a profiling method for analysis of protein kinase expression in cells and tissues [27]; a method for protein kinase identification by two-dimensional electrophoresis in combination with cyanogens bromide (CNBr) digestion of protein kinases [23]; and an analytical method for intracellular protein kinase expression and phosphorylation state [25].

### 3.1 Expression profiling of protein kinases using MicroRotofor/SDS-PAGE

**Fig. 2:**
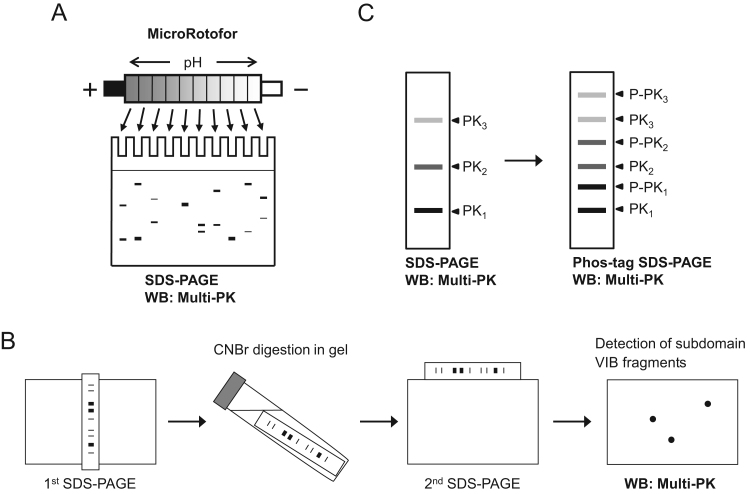
Schematic illustrations of the analytical methods for protein kinases using Multi-PK antibodies. (A) Schematic illustration of the 2D-PAGE analysis for profiling of protein kinases. The expression pattern of the protein kinases was analyzed by Western blotting with Multi-PK antibodies after separation by 2D-PAGE using a MicroRotofor in the first dimension. (B) Outline of the 2D-PAGE analysis to detect subdomain VIB-containing fragments. The procedure consists of the first SDS-PAGE followed by in-gel CNBr digestion, secondary SDS-PAGE, and Western blotting with Multi-PK antibodies. (C) Schematic representation of the phosphorylation state analysis. Western blotting analysis using Multi-PK antibodies was performed after separation by SDS-PAGE (left panel) and Phos-tag SDS-PAGE (right panel). PK: protein kinase; P-PK: phosphorylated protein kinase.

Two-dimensional polyacrylamide gel electrophoresis (2D-PAGE), consisting of isoelectric focusing (IEF) in the first dimension and SDS-PAGE in the second dimension, is the most common technique for analysis of cellular proteins [28]. In recent proteomics studies, 2D-PAGE, in which an immobilized pH gradient (IPG) gel is employed for the first IEF, has been conducted in conjunction with mass spectrometric analysis [29], [30]. However, it was difficult to detect cellular proteins with molecular masses larger than 100 kDa by Western blotting using Multi-PK antibodies after separation by 2D-PAGE. Therefore, we developed a new profiling method for detection of total proteins including high-molecular-mass protein kinases using liquid-phase IEF instead of IPG gel-based IEF in the first dimension (Fig. 2A). MicroRotofor is assembled with 10 sample chambers separated by liquid-permeable screens, such that the proteins can be separated into each compartment without diffusion. When a tissue extract separated by 2D-PAGE using the MicroRotofor was analyzed by Western blotting with Multi-PK antibodies, several proteins with similar sizes, but different p*I* values, were detected in the different lanes [27]. Notably, large proteins with molecular masses above 100 kDa were detected efficiently, indicating that MicroRotofor/SDS-PAGE combined with Western blotting using Multi-PK antibodies is a powerful technique for profiling of cellular protein kinases.

### 3.2 Identification of protein kinases by 2D-PAGE analysis of CNBr fragments of protein kinases

Using Multi-PK antibodies, various protein kinases can be detected in tissue extracts by Western blotting. In recent years, mass spectrometry has been employed for the identification of various unknown proteins in proteomics studies [29], [30]. Although this technique is very useful, we often failed to identify the kinases of interest, probably because there were insufficient amounts of protein in the immunoreactive spots detected by the sensitive antibodies. Therefore, we developed a convenient technique to identify protein kinases without using mass spectrometry.

Among the 518 human protein kinases, only a few have Met residues in their subdomain VIB sequences, as the epitopes for the Multi-PK antibodies. When a crude extract containing various protein kinases was extensively digested with CNBr, immunoreactive fragments containing subdomain VIB were produced. Using the protein kinases sequence data, the molecular sizes of the CNBr-cleaved fragments containing subdomain VIB were estimated. The outline of this method for identifying protein kinases is schematically illustrated in Fig. 2B. After separation of the crude extract by SDS-PAGE, each gel lane is excised and treated with CNBr. The gel strip is then subjected to a second SDS-PAGE, and analyzed by Western blotting with Multi-PK antibodies, allowing estimation of the molecular masses of the CNBr-cleaved fragments of the target protein kinase. Based on the molecular masses of the native protein and the CNBr-cleaved fragments, and the p*I* value, the kinase can be deduced by searching the Swiss-Prot database using the TagIdent program [31]. The identity of the kinase can be validated by Western blotting with a specific antibody against the target kinase. In previous studies, protein kinases such as CaMKIV and DCLK were identified from rat tissue and cell extracts [23], [32], indicating that the method can be applicable to the identification of unknown protein kinases without using mass spectrometry.

### 3.3 Expression and phosphorylation state profiling of protein kinases using Phos-tag SDS-PAGE

The expression and activity of protein kinases play pivotal roles in phosphorylation signaling. Although the expression pattern of protein kinases can be analyzed by Western blotting with Multi-PK antibodies, the activities of protein kinases cannot be assessed by these antibodies. The activity of a large number of protein kinases is regulated by phosphorylation of a critical amino acid residue in an activation loop and/or regulatory domain of the protein kinase by an upstream kinase or autophosphorylation [33]. Therefore, protein kinase activity is closely correlated with the phosphorylation state of the enzyme. To separate phosphorylated proteins from their nonphosphorylated forms, phosphate-affinity SDS-PAGE (Phos-tag SDS-PAGE) has been developed on the basis of mobility shift detection of phosphorylated proteins using a Phos-tag polyacrylamide gel [34], [35], [36]. Recently, we developed a combined method involving Phos-tag SDS-PAGE and Western blotting with Multi-PK antibodies to analyze the expression and phosphorylation state of protein kinases (Fig. 2C).

To detect changes in protein kinase expression and phosphorylation state, human promyelocytic leukemia HL-60 cells were treated with anticancer agents. When the cell extracts were separated by Phos-tag SDS-PAGE and analyzed by Western blotting with Multi-PK antibodies, the immunoreactive band patterns were markedly changed and the band shifts were altered by λPPase treatment [25]. These findings indicate that this unique method can be used to detect alterations in the expression and phosphorylation state of various protein kinases in cells.

## 4 Kinomics studies using Multi‐PK antibodies

### 4.1 Identification of a protein kinase that binds and phosphorylates DNA methyltransferase 1 (Dnmt1)

**Fig. 3:**
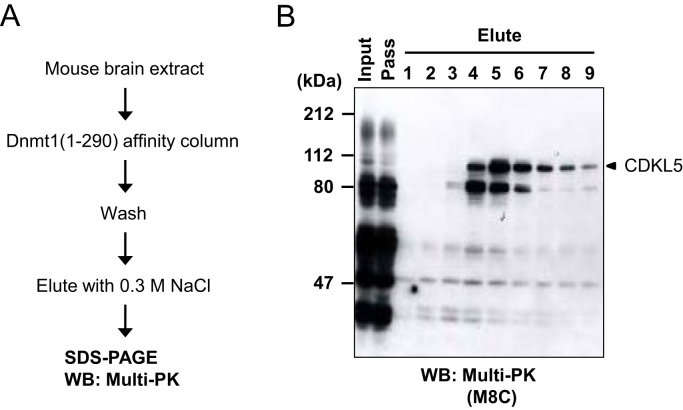
Isolation and identification of Dnmt1-binding kinases. (A) Outline of the isolation of Dnmt1-binding proteins. A mouse brain extract was applied to a Dnmt1(1-290)-affinity column, and the unbound proteins were eluted out with an equilibration buffer. The Dnmt1-binding proteins were eluted with 0.3 M NaCl. (B) The protein kinases in each fraction were analyzed by Western blotting with the M8C Multi-PK antibody. The arrowhead indicates the migration position of CDKL5, which was identified by LC-MS/MS analysis.

DNA methylation is involved in the regulation of gene expression [37], [38]. Although Dnmt1 plays a critical role in maintaining DNA methylation pattern [39], its regulatory mechanisms have not been fully elucidated. The N-terminal region of Dnmt1 forms an independent domain [40], which serves as a regulatory domain with DNA-binding motif [41] and interacts with various proteins such as proliferating cell nuclear antigen and methylated-CpG-binding protein 2 (MeCP2) [42], [43]. Therefore, we attempted to explore the protein kinases that can bind to the regulatory region of Dnmt1. For this experiment, a GST-Dnmt1(1-290) affinity column was used to isolate protein kinases that bound to Dnmt1, and analyzed the bound proteins by Western blotting with Multi-PK antibodies (Fig. 3A). A 110-kDa protein detected in the Dnmt1-binding fractions was identified as CDKL5 by LC-MS/MS analysis (Fig. 3B). CDKL5 could bind to Dnmt1 *in vitro*, and was found to co-localize with Dnmt1 in the nucleus. Furthermore, CDKL5 only phosphorylated Dnmt1 in the presence of DNA [22]. It was reported that defects in the *MeCP2* or *CDKL5* gene caused similar phenotypes to X-linked neurodevelopmental disorders such as Rett syndrome [44]. Consequently, the observed interactions of Dnmt1 with MeCP2 and CDKL5 suggest that epigenetic regulation of gene expression may be involved in the pathogenic processes of Rett syndrome.

### 4.2 Analysis of a protein kinase involved in the pathophysiology of glucotoxicity in type 2 diabetes

Chronically-elevated glucose concentrations have deleterious effects on insulin secretion and expression in pancreatic β-cells [45], [46]. Defective insulin secretion and expression lead to further hyperglycemia and results in glucotoxicity. This negative feedback mechanism accelerates the aggravation of diabetes and the development of diabetic complications [47]. However, the molecular mechanisms of this glucotoxicity are still not fully understood.

**Fig. 4:**
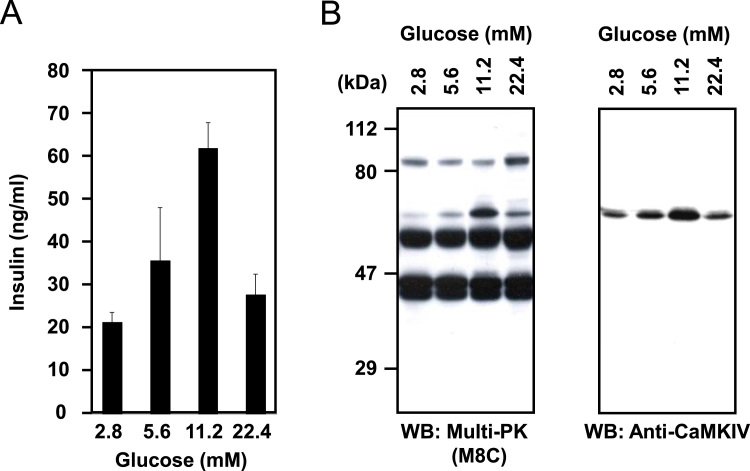
Identification of a protein kinase correlated with insulin secretion. (A) Effect of glucose concentration on basal insulin secretion in INS-1 cells. Secretion of insulin was assessed by sandwich-type ELISA, and the data represent means ± SE (n = 3) of separate experiments. (B) The protein kinases (left panel) and CaMKIV (right panel) in INS-1 cells cultured under varying glucose concentrations were detected by Western blotting using the M8C Multi-PK antibody and an anti-CaMKIV antibody.

When rat insulinoma cell line INS-1 cells, as type 2 diabetes model cells, were cultured in medium containing 2.8–22.4 mM glucose, the basal insulin secretion increased in INS-1 cells cultured with 11.2 mM glucose, but decreased severely at 22.4 mM glucose (Fig. 4A). The expression of protein kinases in INS-1 cells exposed to different glucose concentrations were analyzed using cell extracts by Western blotting with Multi-PK antibodies. As shown in Fig. 4B (left panel), more than five protein kinase bands were detected in INS-1 cells. Notably, the protein level of a 63-kDa immunoreactive band changed in parallel with the change in insulin secretion. To identify the 63-kDa protein kinase, we used the above-described protein kinase identification techniques (shown in Fig. 2B), and found that this kinase was CaMKIV (Fig. 4B right panel). We also obtained evidence that CaMKIV regulated insulin gene expression in INS-1 cells under glucotoxic conditions [32]. These findings suggest that CaMKIV plays important roles in insulin expression under glucotoxic conditions.

### 4.3 Determination of a protein kinase involved in the pathogenesis of ulcerative colitis

Ulcerative colitis and Crohn's disease are known as inflammatory bowel diseases [48]. Although the pathogenesis of inflammatory bowel diseases is regulated by Toll-like receptors activated by intestinal bacteria [49], [50], their etiology is still unclear.

An epithelial cell lysate from oxazolone-administered mice, which showed a similar phenotype to human ulcerative colitis [51], was separated by 2D-PAGE, and analyzed by Western blotting with the YK34 Multi-PK antibody. More than 10 immunoreactive spots were detected, and among them, the expression level of the spot with molecular size of 120 kDa and p*I* value of 6.3 was remarkably increased. Based on these values, the protein was searched using the TagIdent program [31], resulting in several tyrosine kinases, including FAK, being suggested by the search. The spot was subsequently identified as FAK by Western blot analysis using an anti-FAK antibody. The expression level of FAK was found to be correlated with the severity of colitis [26]. These findings suggest that FAK is involved in the pathogenic mechanisms of colitis.

## 5 Conclusions

It is important to develop new technologies and analytical tools to investigate protein kinases, because protein phosphorylation plays crucial roles in diverse biological events. In this review, we have summarized the generation and application of novel monoclonal antibodies, Multi-PK antibodies, directed against various kinase families. The expression patterns of protein kinases in crude cell extracts were analyzed after separation by 2D-PAGE and the phosphorylation states of protein kinases were investigated after Phos-tag SDS-PAGE. The protein kinases involved in particular biological events or pathogeneses of certain diseases were selectively detected and identified using Multi-PK antibodies. Based on the above data, Multi-PK antibodies can be useful tools to analyze not only Ser/Thr protein kinases, but also Tyr protein kinases when used in combination with other conventional technologies or analytical tools such as kinase-specific antibodies or phosphorylation site-specific antibodies. We expect that Multi-PK antibodies will become powerful tools to open a new protein kinase world.
